# MitoAge: a database for comparative analysis of mitochondrial DNA, with a special focus on animal longevity

**DOI:** 10.1093/nar/gkv1187

**Published:** 2015-11-20

**Authors:** Dmitri Toren, Thomer Barzilay, Robi Tacutu, Gilad Lehmann, Khachik K. Muradian, Vadim E. Fraifeld

**Affiliations:** 1 The Shraga Segal Department of Microbiology, Immunology and Genetics, Center for Multidisciplinary Research on Aging, Ben-Gurion University of the Negev, Beer-Sheva, Israel; 2 Tumor and Vascular Biology Research Center, The Rappaport Faculty of Medicine and Research Institute, Technion – Israel Institute of Technology, Haifa, Israel; 3 Life Span Prolongation Group, Institute of Gerontology, Kiev, Ukraine

## Abstract

Mitochondria are the only organelles in the animal cells that have their own genome. Due to a key role in energy production, generation of damaging factors (ROS, heat), and apoptosis, mitochondria and mtDNA in particular have long been considered one of the major players in the mechanisms of aging, longevity and age-related diseases. The rapidly increasing number of species with fully sequenced mtDNA, together with accumulated data on longevity records, provides a new fascinating basis for comparative analysis of the links between mtDNA features and animal longevity. To facilitate such analyses and to support the scientific community in carrying these out, we developed the MitoAge database containing calculated mtDNA compositional features of the entire mitochondrial genome, mtDNA coding (tRNA, rRNA, protein-coding genes) and non-coding (D-loop) regions, and codon usage/amino acids frequency for each protein-coding gene. MitoAge includes 922 species with fully sequenced mtDNA and maximum lifespan records. The database is available through the MitoAge website (www.mitoage.org or www.mitoage.info), which provides the necessary tools for searching, browsing, comparing and downloading the data sets of interest for selected taxonomic groups across the Kingdom Animalia. The MitoAge website assists in statistical analysis of different features of the mtDNA and their correlative links to longevity.

## INTRODUCTION

Mitochondria are the only organelles in the animal cells that have their own genome. The stability of the mitochondrial DNA (mtDNA) is vital for mitochondrial proper functioning; therefore, changes in mtDNA may have far-reaching consequences for the cell fate and, ultimately, for the whole organism. Not surprisingly, due to a key role in energy production, generation of damaging factors (ROS, heat), and regulation of apoptosis, mitochondria and mtDNA in particular have long been considered one of the major players in the mechanisms of aging, longevity and age-related diseases (1–6).

Mitochondrial DNA exists in multiple copies and typically contains genes encoding for 13 key subunits of the respiratory chain enzymes, a set of 22 tRNA genes, and 2 genes for the large (16S) and small (12S) rRNA subunits. In contrast to the nuclear DNA, mtDNA is a circular, intronless, extremely compact molecule, with asymmetric distribution of nucleotides between the heavy (G-rich) and light (C-rich) strands (7,8). With very few exceptions, such structure of mtDNA is typical for the vast majority of animal species.

Longevity (generally estimated by maximum lifespan, MLS) varies greatly among animal species (9) (genomics.senescence.info/species). Species also differ in their mtDNA compositional features (10), which to a great extent may determine the mtDNA stability and mutability. A few lines of evidence point towards a putative significance of mtDNA in aging and longevity. Firstly, mtDNA mutations accumulate with advanced age (5,11,12). Secondly, strong correlative links between mammalian MLS and mtDNA compositional features have been found (4,6,13–18).

The rapidly increasing number of species with fully sequenced mtDNA genomes, together with accumulated data on longevity records, provide now a strong basis for comprehensive comparative analysis of the links between mtDNA features and animal longevity. Yet, efficient processing of such amount of data is computationally demanding. In turn, this generates a need for appropriate databases and bioinformatics tools. With the creation of MitoAge, we aim to encourage the modeling of mtDNA-longevity relationships, providing the scientific community with one single place to access, compare and analyze the data, based on most updated resources. MitoAge (www.mitoage.org, www.mitoage.info) is a curated, publicly available database, which contains an extensive repository of mtDNA data integrated with longevity records and the results of the statistical and correlative analysis of the links between them.

## DATABASE CONTENT AND INTERFACE

To date, 5337 entries with complete mitochondrial genomes and 4237 entries with longevity records are available at NCBI RefSeq database and the AnAge database, respectively (see the Data Sources section). The overlap of these two data sets after curation, encompassing 922 animal species covering 304 families, 106 orders and 13 classes from the Kingdom Animalia, was included in MitoAge, Build 1.0. As seen in Table 1, the vast majority of species are vertebrates, with only few representatives of invertebrates.

**Table 1.** Number of species per taxon in MitoAge

Taxa (scientific name)	Species
Mammals (Mammalia)	390
Birds (Aves)	152
Reptiles (Reptilia)	94
Amphibians (Amphibia)	29
Fishes (Actinopterygii, Sarcopterygii, Cephalaspidomorphi, Chondrichthyes)	251
Non-chordates (Bivalvia, Echinoidea, Chromadorea, Insecta, Malacostraca)	6

MitoAge contains compositional features (base content, GC%, AT%, sequence length) of the entire mitochondrial genome, mtDNA coding (tRNA, rRNA, protein-coding genes) and non-coding (D-loop) regions for each species and taxonomic group. For protein-coding genes of a given species, codon usage with distribution of codons by base position (e.g. codons with first base G, C, A or T) and amino acids frequencies are included. Along with mtDNA data, longevity records (MLS) are presented.

Additionally, the MitoAge database tools provide the user with a number of options for (i) computation of basic statistics (range, median, mean ± standard deviation, coefficient of variation, Pearson's coefficient of correlation with log-transformed MLS); (ii) comparison of stats between selected taxonomic groups (two or more); (iii) data export for a data set of interest (in a CSV format), without downloading the entire database. If a user needs a more complex analysis, the website allows downloading the entire database, which can be done from the Download page in versioned releases (numbered database builds).

**Figure 1.**
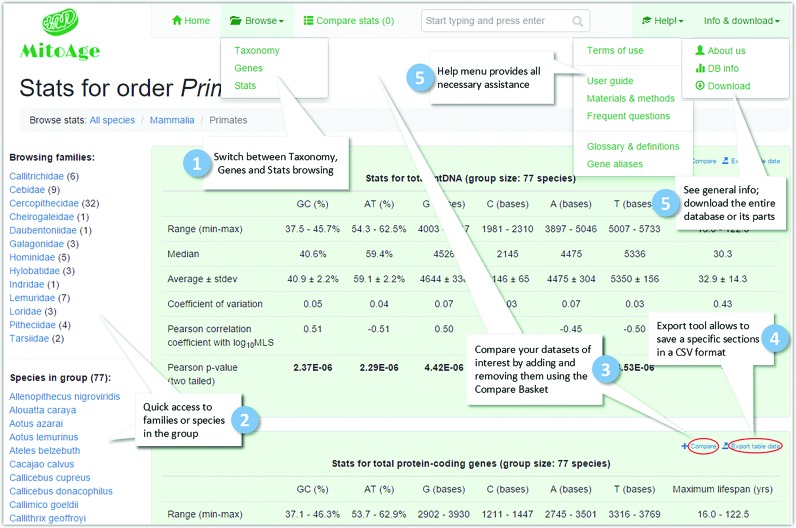
A labeled diagram of the MitoAge interface.

**Figure 2.**
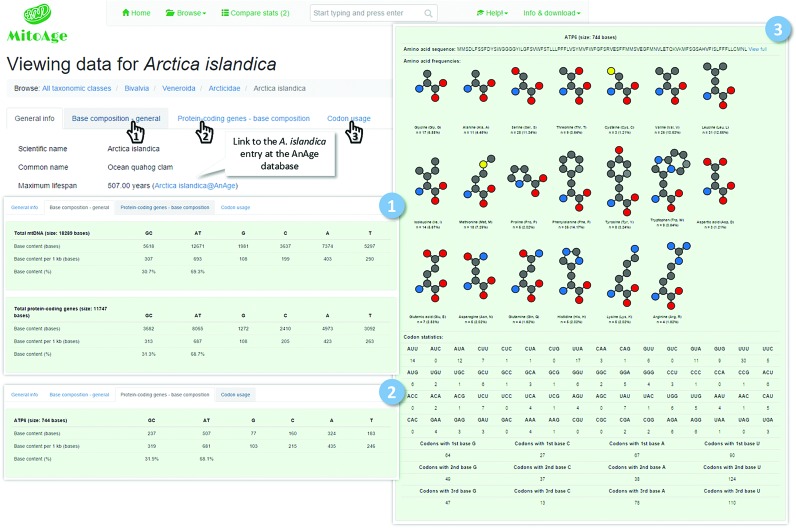
Example: viewing data for *Arctica islandica*.

MitoAge has a user-friendly website interface with simple and intuitive navigation tools (Figures 1 and 2). *Searching* can be done either by species common or scientific name, or by taxonomy groups (i.e. by classes, orders or families). Alternatively, the data can be reached by *Browsing* in three different ways: (i) Browsing *Taxonomy* (classes, orders, families or species); (ii) Browsing *Stats*, which calculates on-the-fly statistical information for the total mtDNA or specific genes/regions for the selected taxonomic group; (iii) Browsing *Genes*, an option similar to that of browsing stats, but providing data restricted to a gene of interest.

## DATA SOURCES AND DATA CURATION

The MitoAge database was constructed using publicly available data, is being constantly updated through automatic tools and is manually curated for problematic issues.

Complete mtDNA sequences were taken from NCBI RefSeq database (www.ncbi.nlm.nih.gov/refseq) (19); longevity records were retrieved from the HAGR: Human Ageing Genomic Resources—AnAge database (genomics.senescence.info/species) (9), and full taxonomy data were retrieved from the Integrated Taxonomic Information System (ITIS) on-line database [31-Jul-2015 Build] (www.itis.gov).

Most of the data included in the MitoAge database were computed offline, using a series of automated scripts and programs developed in our lab, with a number of parameters computed on-the-fly through the website. Together with a series of administration tools, this ensures frequent updates of the database.

Data were computed and analyzed as follows: (i) base composition and size were generated for total mtDNA and its specific regions/genes (D-loop, protein-coding genes, rRNA-coding genes and tRNA-coding genes); (ii) for each protein-coding gene and for the total protein-coding sequence, both base composition and codon usage/amino acids frequency were computed.

All data available at MitoAge have undergone a three-step validation: (i) the collected raw data from NCBI RefSeq was processed through our software and log files were generated; (ii) reports of errors and inconsistencies from log files were then manually curated; and finally (iii) additional tests for database consistency (e.g. duplicates, faulty values, etc.) were performed by the administrative tools in the MitoAge website. The list of errors and inconsistencies found in the mtDNA sequence annotations were reported to the NCBI admin and subsequently corrected by the NCBI team. Upon successful validation, the corrected data were uploaded into the MitoAge database and various statistical metrics were computed.

## DATA CALCULATION

The compositional features of the mtDNA heavy strand (H-strand) were mostly considered (unless indicated otherwise), since the H-strand represents a primary target for the directional mutation pressure (20). For computing codon usage, data were taken from the complement of the coding strand and Thymine (T) was replaced with Uracil (U) (i.e. transforming it into the mRNA sequence). When combining multiple genes (e.g. when computing the total protein-coding genes), we append one gene to another and use the entire sequence for the analysis. In case of overlap between genes, we count the overlapping sequences only once for the computation of base composition, and we count them twice for the computation of codon usage and amino acids frequencies for each gene. Sequences are always analyzed from 5′ to 3′ (both for DNA and RNA).

## AVAILABILITY

The MitoAge database is available at www.mitoage.org and www.mitoage.info, with the data made available under the permissive Creative Commons license, allowing data to be used in other analyses. There are options to either download the entire database or its parts, or to export specific results of the website. Feedback via email is welcome.

## CONCLUSION

The MitoAge database is an integrated web resource for comparative analysis of mtDNA, with a special focus on animal longevity. Thus, it fills the gap in one of the ‘hot spots’ at the crossroad of aging research, evolutionary and mitochondrial biology. MitoAge novelties, together with a user-friendly interface, provide unique capabilities for in-depth investigation of: (i) the abundance of mtDNA bases and its variability across animal taxa with different MLS; (ii) the links between longevity and the mtDNA compositional features (total, region or gene-specific), including codon usage and amino acids frequency. Using the MitoAge database, we revealed more than 3780 statistically significant correlations between mtDNA features and animal longevity in different taxonomic groups. MitoAge could be also a useful supporting tool for building predictive models of animal longevity, involving mtDNA features.

## FUNDING

Fund in Memory of Dr Amir Abramovich. Funding for open access charge: Ben-Gurion University of the Negev [to V.E.F.].

*Conflict of interest statement*. None declared.
